# Multiple Colonization with *S. pneumoniae* before and after Introduction of the Seven-Valent Conjugated Pneumococcal Polysaccharide Vaccine

**DOI:** 10.1371/journal.pone.0011638

**Published:** 2010-07-16

**Authors:** Silvio D. Brugger, Pascal Frey, Suzanne Aebi, Jason Hinds, Kathrin Mühlemann

**Affiliations:** 1 Institute for Infectious Diseases, University of Bern, Bern, Switzerland; 2 Division of Cellular and Molecular Medicine, St. George's, University of London, London, United Kingdom; 3 Department of Infectious Diseases, University Hospital, Bern, Switzerland; Charité-Universitätsmedizin Berlin, Germany

## Abstract

**Background:**

Simultaneous carriage of more than one strain of *Streptococcus pneumoniae* promotes horizontal gene transfer events and may lead to capsule switch and acquisition of antibiotic resistance. We studied the epidemiology of cocolonization with *S. pneumoniae* before and after introduction of the seven-valent conjugated pneumococcal vaccine (PCV7).

**Methodology:**

Nasopharyngeal swabs (n 1120) were collected from outpatients between 2004 and 2009 within an ongoing nationwide surveillance program. Cocolonization was detected directly from swabs by restriction fragment length polymorphism (RFLP) analysis. Serotypes were identified by agglutination, multiplex PCR and microarray.

**Principal Findings:**

Rate of multiple colonization remained stable up to three years after PCV7 introduction. Cocolonization was associated with serotypes of low carriage prevalence in the prevaccine era. Pneumococcal colonization density was higher in cocolonized samples and cocolonizing strains were present in a balanced ratio (median 1.38). Other characteristics of cocolonization were a higher frequency at young age, but no association with recurrent acute otitis media, recent antibiotic exposure, day care usage and PCV7 vaccination status.

**Conclusions:**

Pneumococcal cocolonization is dominated by serotypes of low carriage prevalence in the prevaccine era, which coexist in the nasopharynx. Emergence of such previously rare serotypes under vaccine selection pressure may promote cocolonization in the future.

## Introduction

The epidemiology of *Streptococcus pneumoniae* is changing since the introduction of the seven-valent conjugated pneumococcal polysaccharide vaccine (PCV7) [1], [2], [3], [4]. Non-vaccine serotypes are emerging and some new clones have evolved by capsule switch [5], [6], [7], [8]. Current evolution of *S. pneumoniae* under vaccine selection pressure demonstrates the plasticity of this pathogen, but the direction and consequences of such evolution cannot be predicted. Evolution of *S. pneumoniae* occurs by lateral gene transfer in the human nasopharynx, the natural habitat of pneumococci [9], [10]. Gene transfer between pneumococcal strains likely requires the simultaneous presence of two pneumococcal strains in the nasopharynx also termed multiple colonization or cocolonization.

Data about the epidemiology of multiple colonization are limited. Existing prevalence estimates range from 1.3% to 30% [11], [12], [13], [14], [15], [16], [17], [18]. Geographical variations in pneumococcal epidemiology and different techniques employed may be responsible for such variation. Studies on cocolonization have been hampered by the lack of sensitive, economic and easy to apply methods for the detection of multiple strains in nasopharyngeal samples. Recently, we reported on a new molecular method for the detection of multiple colonization [19]. In this study we applied this technique to a large collection of nasopharyngeal swabs (n 1120) collected before and after the introduction of PCV7 in Switzerland in 2006. This report presents novel information about the epidemiology, determinants and time trends of multiple colonization with *S. pneumoniae* in the vaccine era. We hypothesized that reduction of the prevalent serotypes included in PCV7 and emergence of new serotypes may affect the rate of cocolonization and the strains or serotypes involved in cocolonization.

## Materials and Methods

### Ethic statement

Nasopharyngeal swabs used for this study were collected within a nationwide surveillance program from outpatients with acute otitis media or pneumonia, which is ongoing since 1998 [20], [21]. This surveillance program is part of the governmental public health surveillance and is therefore exempted from approval by Institutional Review Boards.

### Collection of nasopharyngeal swabs

For this study swabs from four winter seasons between 2004 and 2009 were analyzed. Period 1 spanned from December 2004 to February 2005, period 2 from November 2006 to March 2007, period 3 from November 2007 to March 2008 and period 4 from November 2008 to March 2009. Parts of the data from period 1 have been presented earlier [19]. In Switzerland, the seven-valent conjugated pneumococcal polysaccharide vaccine (PCV7) has been recommended since May 2006 to all children under the age of 24 months in a three dose schedule given at 2, 4 and 12 months and since August 2006 the vaccine is reimbursed by health insurance.

### Diagnostic work-up of nasopharyngeal swabs

Processing of swabs for culture and capsular serotyping of *S. pneumoniae* and for direct molecular detection of pneumococcal cocolonization by *plyNCR* PCR and subsequent restriction fragment length polymorphism (*plyNCR* RFLP) and terminal-RFLP (T-RFLP) has been described in detail previously [19], [22]. Briefly, swabs were streaked out onto a CSBA (Columbia sheep blood agar) plates and were then eluted in 800 µl of either transport medium for chlamydia and viruses (TMCV, [19]) or in water. The swab solution was stored at −80°C until further use. All pneumococcal isolates detected by primary conventional culture were subcultured once on CSBA plates and stored at −80°C (Protect®, Technical Service, Lancashire, UK). In addition, during study period 4 the entire bacterial lawn grown on the primary CSBA culture plate was collected and stored at −80°C (Protect®, Technical Service, Lancashire, UK).

### Detection of pneumococcal colonization and multiple colonization by *plyNCR* PCR, RFLP and T-RFLP

Chromosomal bacterial DNA was isolated from stored swab solution and *plyNCR* PCR was performed as described previously [19]. In order to estimate the amount of pneumococcal DNA in swab samples as a surrogate for colonization density chromosomal DNA of three clinical isolate of *S. pneumoniae* (strain B109.48 of serotype 9V, strain 109.24 of serotype 19F, strain 312.57 of serotype 3) was isolated [23] and DNA concentration was determined with a Nanodrop ND-100 spectrophotometer (Nanodrop Technologies, Wilmington, DE). DNA was then diluted with double distilled water to amounts of 100, 10, 1, 0.1, 0.01 and 0.001 ng. After *plyNCR* PCR amplification DNA concentration was determined using the Agilent 2100 bioanalyzer (Agilent Technologies, Palo Alto, CA). A standard curve was generated by plotting PCR-product concentration against amount of DNA. This showed a linear relationship for the range of bacterial DNA concentrations usually found in nasopharyngeal swabs (data not shown). *plyNCR* RFLP analysis was performed as described in [19] and samples for which the sum of the size of the bands obtained after restriction enzyme digestion was greater than the size of the undigested PCR product were further analyzed for cocolonization. In order to confirm the presence or absence of *S. pneumoniae* in samples with *plyNCR* PCR products of atypical band size or >1 band, PCR for *psaA* was performed as described [19], [24].

T-RFLP analysis was used to determine the number of strains and their relative quantity in cocolonized samples as described except that capillary electrophoresis was performed on an ABI Genetic Analyzer 3130 for samples in time periods 2 to 4 [19]. In addition, T-RFLP with the two enzymes BtsCI and Bsu36I was used to detect cocolonization in samples with low pneumococcal DNA content.

### Detection of capsular serotypes

Capsular serotypes were determined for cultured isolates by the “agglutination reaction” (Statens Serum Institute, Copenhagen, Denmark). Multiplex PCR and microarray were employed for molecular detection of serotypes in cocolonized samples. Both techniques are usually not performed directly on swab material, due to lower sensitivity and requirement of large amounts of swab material. Therefore, we used frozen stocks from primary culture isolates and frozen stocks from bacterial lawns from the primary culture plate for these tests. Multiplex PCR for 29 capsular serotypes/serogroups was performed according to the method described by Pai et al. [25] (except that PCR analysis was performed with the Agilent bioanalyzer). Molecular serotyping by microarray was performed using the BμG@S SP-CPSv1.3.0 microarray using standard protocols. Briefly, DNA samples were fluorescently labelled and hybridized to the Agilent 8×15K format microarray according to manufacturer's instructions for the Agilent genomic DNA enzymatic labelling and oligo aCGH hybridisation reagent kits. Microarray data was statistically analysed using a Bayesian hierarchical model (Newton *et al.,* unpublished data) to determine the serotype, or combination of serotypes, present in the sample and assign a relative abundance of each serotype detected.

### Statistical analysis

Statistical analysis was done in StatView® version 5.0 (SAS Institute Inc., Cary, NC). Differences between means were tested by the Student's T-test or the Mann-Whitney U test and proportions were compared with the Chi Square or Fisher's exact test, as appropriate. Risk factors identified by univariate statistics were entered into ANOVA or a logistic regression model as appropriate. The final model contained the largest number of variables with p ≤0.05. A cut-off of p ≤0.05, two tailed, was used for all statistical analyses.

## Results

### Pneumococcal carriage rate by culture and PCR

The overall pneumococcal carriage rate was 45.8% (514 of 1120 swabs) by culture or PCR, the rate was 39.7% by bacterial culture, and 45.6% by PCR. Sixty nine samples (6.1%) were positive by PCR only and three samples were positive by culture only. A total of 389 of 511 (74.3%) PCR positive samples were screened for cocolonization by *plyNCR* RFLP and T-RFLP. The remaining 122 samples could not be processed, because quantity of DNA recovered from swabs was insufficient for enzyme digestion (n = 83), or because *ply*NCR PCR amplified more than one band (n = 40), which inhibited interpretation of restriction enzyme patterns [19].

### Pneumococcal vaccination rates

Patients were mostly children and about half of patients were below the age of 5 years (49.0%, n 549). PCV7 vaccination rates increased during the study period. Among children less than 5 years of age vaccination rates were 3.2% for study period 1, 33.3% for study period 2, 50% for study period 3, and 77.8% for study period 4. The corresponding rates for children <2 years of age for time periods 1, 2, 3, and 4 were 1.4%, 47.3%, 76.3%, and 88.4%, respectively. Therefore, study period 1 was considered the “prevaccine era” and the later seasons combined were defined as the “vaccine era”.

### Detection of multiple colonization

**Figure 1:**
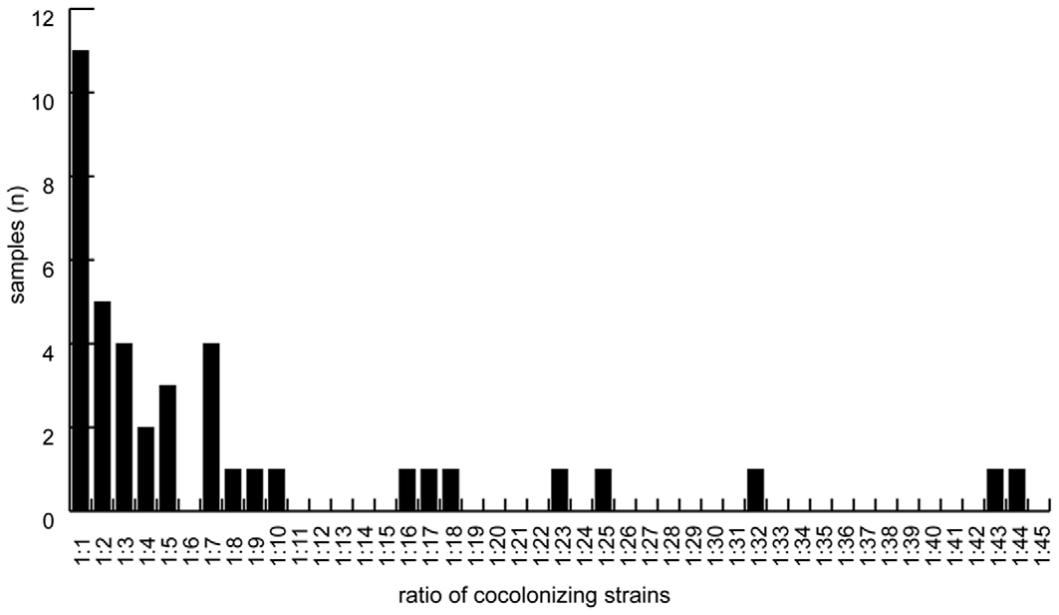
Ratio of strains present in cocolonized nasopharyngeal swabs. Strain ratios were determined from the peak heights in the chromatograms obtained by terminal-restriction fragment length polymorphism analysis (T-RFLP, as described in the methods section). The X-axis indicates the fold-higher presence of the more abundant strain. The three samples with three strains were excluded.

Cocolonization was detected in 41 nasopharyngeal swabs by RFLP or T-RFLP. Only seven of these 41 cocolonization events were also detected by conventional serotyping of multiple (n = 20) colonies from primary swab culture plates. Two different strains were present in 38 of the 41 samples and three samples contained three strains. The relative ratio of strains present in the same sample ranged from 1∶1 to a maximum of 1∶45, with a median ratio of 1∶3.8 (Figure 1).

### Frequency of multiple colonization

The rate of cocolonization was 3.6% among all samples (n 1120) and 7.9% among samples with detection of *S. pneumoniae* (n 514). The rate of cocolonization was similar before and after introduction of PCV7 with a prevaccine rate of 3.4% (10 of 287 samples) or 6.7% (10 of 149 samples with detection of *S. pneumoniae*) as compared to 3.7% (31 of 833 samples) or 8.4% (31 of 365 samples with detection of *S. pneumoniae*) after introduction of PCV7. Cocolonization rates were also stable over the four time periods analysed (data not shown).

### Factors associated with multiple colonization

Cocolonization was more frequent at younger age (Table 1). The probability of cocolonization was not affected by recent antibiotic treatment, day care use, a history of recurrent acute otitis media or PCV7 vaccination status. However, pneumococcal colonization density (the concentration of the *plyNCR* PCR product) was significantly higher in samples with multiple colonization (p<0.001) (Table 2).

**Table 1:** Univariate analysis for factors associated with carriage of single or multiple strains of *Streptococcus pneumoniae*.

	Cocolonization		Single colonization		Not colonized		p
	N	%	N	%	N	%	
Total	41	100	473	100	606	100	
Introduction of PCV7^a^							
prevaccine era	10	25.6	139	29.4	138	22.8	
vaccine era	31	74.4	334	70.6	468	77.2	
Age (years)^b^							
<2	14	34.1	138	29.2	119	19.8	<0.001
2–4	14	34.1	150	32.0	113	18.8	
5–16	11	26.8	117	24.8	144	24.0	
17–64	2	4.9	55	11.7	170	28.3	
>64	0	0	11	2.3	54	9.0	
Female gende^c^	17	41.5	207	44.0	286	47.6	0.43
Antibiotic treatment^d^	8	20.5	55	12.9	84	15.4	0.29
Day care^e^ in children <5 years old	7	26.9	106	39.5	72	33.6	0.24
Recurrent otitis^f^ media in children <5 years old	4	15.4	55	21.2	47	22.3	0.71
PCV7 vaccinated^g^ in children <5 years old	9	34.6	102	36.3	98	45.4	0.03

a PCV7 = 7-valent conjugated pneumococcal polysaccharide vaccine.

b No information was available about age for 7 patients.

c No information was available about gender for 4 patients.

d Antibiotic treatment during the 8 weeks before swab collection, no information was available for 108 patients (9.6%).

e No information about current day care use was available for 152 patients (13.5%).

f More than one otitis media episode during the past 12 months. No information was available about recurrent otitis media for 183 patients (16.3%).

g Vaccination with the seven-valent conjugated pneumococcal vaccine.

No information about vaccination status was available for 100 patients (8.9%). P-value is for the comparison between carriage of single or multiple strains of *S. pneumoniae* versus no carriage.

**Table 2:** Univariate analysis of DNA quantity of *plyNCR* PCR products as a surrogate for pneumococcal colonization density in the nasopharynx.

	DNA concentration of PCR product					
	ng/µl			Proportion of samples with <10 ng/µl		
	mean	SD	p	N	%	p
All	26.6	20.2		149	31.7	
Age (years)^a^			0.07			0.03
<2	29.1	20.5		37	24.5	
2–4	27.2	19.4		44	26.9	
≥5	24.3	20.5		68	34.8	
Gender			0.02			0.04
Female	29.0	20.3		54	24.2	
Male	25.0	19.9		92	32.2	
PCV7 vaccinated^b^, <2 years olds			0.06			0.08^c^
yes	26.1	20.0		22	30.9	
no	32.4	20.8		14	18.6	
Cocolonization			0.003			<0.001
yes	35.3	16.5		0	0	
no	25.9	20.3		149	31.7	
PCV7 serotype^d^, <2 years olds			0.20			0.92
yes	38.0	19.0		5	12.1	
**no**	32.6	19.9		7	16.2	

a No information was available about age for 7 patients.

b PCV7 =  seven-valent conjugated pneumococcal vaccine.

c This associations became statistically significant after adjustment for carriage of a serotype contained in PCV7 in a logistic regression model (p = 0.02).

d Carriage of a serotype contained in the seven-valent conjugated pneumococcal vaccine; i.e. one of the following serotypes: 4, 6B, 9V, 14, 18C, 19F.

No information about vaccination status was available for 100 patients (8.9%). P-values compare the carriage of single or multiple strains of *S. pneumoniae* to no carriage.

### Multiple colonization and *plyNCR* RFLP-types

We have previously shown that *plyNCR* RFLP can be used for molecular typing of pneumococcal strains [22]. We were interested to analyze whether strains involved in cocolonization belonged to distinct RFLPtypes. A RFLP-type could be determined for 81 of the 85 pneumococcal strains present in samples with multiple colonization. The 81 strains grouped into 30 different RFLP types, of which 13 occurred at a relative frequency of ≥2% relative to all RFLP types detected in this study. The remaining RFLP types with a prevalence of <2% were designated “rare” and were grouped together into the category “others” for analysis. RFLP type 15 (p 0.01), type 17 (p 0.02), and type 19 (p 0.03) were significantly more frequent among cocolonized samples, and samples with only one strain had a higher proportion of type 2 (p 0.02) and type 11 (p 0.01). There were no time trends of RFLP type distribution between the prevaccine and vaccine era (data not shown). Pairs of RFLP types in cocolonized samples showed no predominance of a certain combination of RFLP-types (data not shown).

### Multiple colonization and capsular serotypes

**Figure 2:**
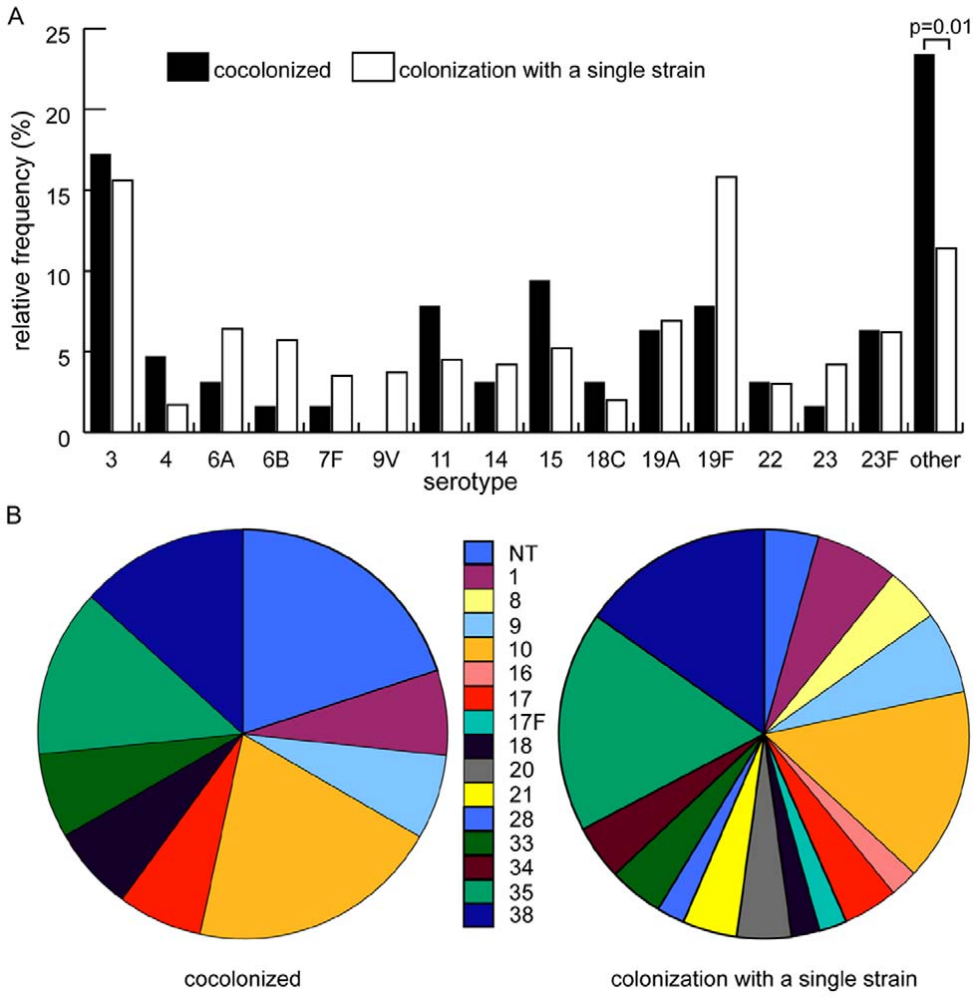
Serotype distribution among nasopharyngeal *S. pneumoniae* isolates. **A.** Serotype distribution among nasopharyngeal isolates (n 468) of *S. pneumoniae* stratified according to the detection of cocolonization or single colonization with *S. pneumoniae* strains. The group “cocolonizing” includes the 64 isolates from cocolonized samples, for which a serotype could be determined. Serotypes with a relative frequency of less than 2% are summarized as “others”. They included serotypes/-groups 1, 8, 9 (except 9V), 10, 16, 17, 17F, 18 (except 18C), 20, 21, 28, 33, 34, 35, 38 and non-typeable isolates. The rate of rare serotypes (“others”) was significantly higher in cocolonized than in single colonized swabs (23.4% vs. 11.4%, p = 0.01), but difference in the frequency of individual serotypes (for example for the serotypes 11, 15, and 19F) did not reach statistical significance. **B.** Distribution of rare serotypes (group “other” in Figure 2A) in samples with or without cocolonization. The difference between the proportion of non-typeable strains in cocolonized samples (4.7%) and in those with a single strain (0.5%) was statistically significant (Fisher's exact test p = 0.02).

A serotype could be determined for 64 of 85 strains detected in the 41 cocolonized samples. The 64 strains grouped into 30 different serotypes and non-typeable pneumococci. Serotypes with a relative frequency of less than 2% were designated “rare” and grouped together as “other serotypes” for analysis. These rare serotypes/-groups included 1, 8, 9 (except 9V), 10, 16, 17, 17F, 18 (except 18C), 20, 21, 28, 33, 34, 35, 38 and non-typeable isolates. Interestingly, in cocolonized samples rare serotypes were more frequently represented than the prevalent serotypes (23.4% versus 11.4%. p = 0.01) (Figure 2A). Also, non-typeable pneumococci were detected more often in cocolonized samples than in those with a single strain (4.7% versus 0.5%, Fisher's exact test p = 0.02) (Figure 2B).

**Figure 3:**
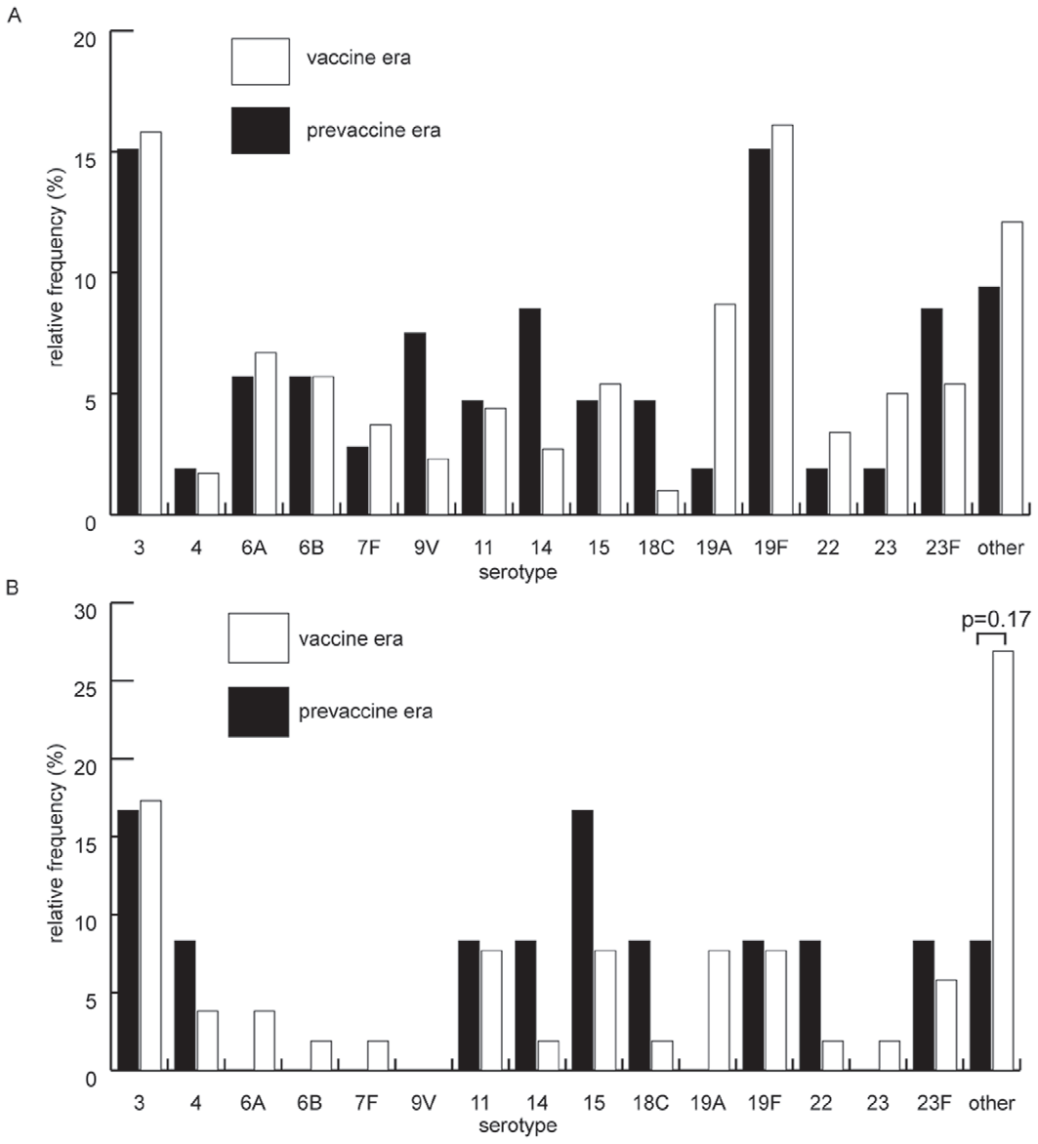
Relative distribution of pneumococcal serotypes among 468 pneumococcal strains isolated in prevaccine or vaccine era. The relative frequency of pneumococcal serotypes isolated from nasopharyngeal swabs before and after the introduction of the seven-valent conjugated polysaccharide vaccine for (**A**) swabs with one serotype only (prevaccine era n 106, vaccine era n 298), and (**B**) restricted to isolates from cocolonized samples (prevaccine era n 12, vaccine era n 52). For easier reading of the graph rare serotypes (prevalence <2%) were grouped as “others”. They included serotypes and/or serogroups 1, 8, 9 (except 9V), 10, 16, 17, 17F, 18 (except 18C), 20, 21, 28, 33, 34, 35, 38 and non-typeable isolates.

Time trends of serotype distribution between the prevaccine and vaccine era were comparable among samples with single or multiple colonization except that the proportion of rare serotypes increased among samples with multiple colonization in the vaccine era (Figure 3).

Pairs of serotypes in cocolonized samples showed no predominance of a certain combination of serotypes, but none of the cocolonized samples showed a combination of serotypes included in PCV7 (data not shown).

## Discussion

Simultaneous colonization of the human nasopharynx with more than one strain or serotype of *S. pneumoniae* is an intriguing event. It sets the stage for pneumococcal evolution through horizontal exchange of genes, which may result in the acquisition of antibiotic resistance or capsule switch [9]. In addition, the study of cocolonization offers insight into the interaction between different strains or serotypes of *S. pneumoniae*. This study shows that PCV7 selection pressure did not affect the cocolonization rate up to three years after the introduction of the vaccine. Therefore, opportunities for horizontal gene transfer between pneumococci in the nasopharynx will continue to occur at the same frequency in the vaccine era as in the prevaccine era and new strains may emerge under vaccine selection pressure as has been described [5].

One might have expected that cocolonization would involve more frequently the prevalent serotypes with high incidence of acquisition and long duration of carriage [21], [26], [27]. However, in this study, rare serotypes predominated among cocolonizing pneumococcal strains. For example, serotype 19F, one of the most carried serotypes, was relatively rare among cocolonizing strains already in the prevaccine era and serotype 19A did not emerge among cocolonizing strains in the vaccine era as it did among single colonizing strains. The observed predominance of rare serotypes during cocolonization suggests that simultaneous colonization is not simply a consequence of cumulative exposure to transmission opportunities. This hypothesis is also supported by the observed lack of an association of cocolonization with day care usage, a history of recurrent otitis media and recent antibiotic exposure.

It has been shown that pneumococcal serotypes differ for their competitiveness in colonizing the nasopharynx [28]; some serotypes, such as 6B can outcompete others, such as 23F. In our study, colonization density was significantly higher in nasopharyngeal swabs containing multiple pneumococcal strains as compared to those swabs with a single strain. Also, the median ratio of two pneumococcal strains in a given sample was balanced (median 1.3). Therefore, cocolonizing strains seem to co-exist rather than to compete with each other for example for resources (i.e. nutrients, space) or by production of bacteriocins. Margolis and colleagues recently showed in a rat colonization study that sequential cocolonization with two pneumococcal strains can lead to stable co-existence and a higher density for cocolonization compared to single colonization [29]. Whether the tendency for co-existence between some pneumococcal serotypes, as described here, may also apply to co-existence with other pathogens such as *H. influenzae* and *S. aureus* needs to be studied [30], [31], [32], [33].

Serotypes, which predominated in multiple colonization in this study, did not include serotypes covered by the seven-, ten- or thirteen-valent conjugated polysaccharide vaccines, and the prevalence of these rare serotypes increased in the vaccine era (Figure 3). Therefore, we can not exclude that rates of multiple colonization may increase in the future with the emergence of these serotypes. The follow-up period of three years after introduction of PCV7 may have been too short to observe such a trend.

A limitation of this study is that the study population was restricted to patients with acute otitis media or pneumonia. The conclusions drawn from this study may therefore not apply to healthy persons. Also, the epidemiology of cocolonization may be different in populations with overall higher carriage rates than observed in our study [11], [17], [34]. Multiplex PCR for the detection of pneumococcal serotypes has also been used for the detection of cocolonization [11]. Whilst multiplex PCR for serotype cannot detect cocolonization by different pneumococcal strains of the same serotype the method used in this study, *plyNCR* RFLP and T-RFLP, will miss cocolonization by two serotypes with an identical RFLP type. We compared the performance of multiplex PCR and T-/RFLP for the detection of cocolonization in 141 consecutive nasopharyngeal swabs (from time period 4) and found a slightly higher sensitivity for the T-/RFLP assay than multiplex PCR: cocolonization was detected by both methods in six samples, by T-/RFLP only in 8 samples, and by multiplex PCR only in one sample (Table 3). Therefore, we believe that this study did not underestimate the cocolonization rate in comparison to studies using multiplex PCR.

**Table 3:** Comparison between multiplex PCR and *plyNCR* RFLP/T-RFLP for the detection of multiple colonization in 141 nasopharyngeal swabs collected during study period 4 for which culture revealed growth of a pneumococcal strain.

Multiple colonization by	Multiple colonization by *plyNCR* RFLP and T-RFLP	
multiplex PCR	Yes	No
Yes	6	1
No	8	126

Multiplex PCR was performed on DNA extracted from frozen stocks of the primary culture plate. *plyNCR* RFLP and T-RFLP detected more episodes of multiple colonization than multiplex PCR positive and In 3 of the 8 cocolonization events not detected by multiplex PCR non-typeable pneumococcal strains were involved.

In summary, this study showed that pneumococcal cocolonization rates have not been affected by the selection pressure of PCV up to 3 years after the introduction of universal vaccination. Multiple colonization involves more often serotypes that have been of low prevalence in the prevaccine era, but may now emerge under vaccine selection pressure. This may lead to an increase in the cocolonization rate in the future, with an increasing opportunity for pneumococcal evolution through horizontal gene exchange.
